# Periodontitis and edentulism as risk indicators for mortality: Results from a prospective cohort study with 20 years of follow‐up

**DOI:** 10.1111/jre.13061

**Published:** 2022-10-25

**Authors:** Georgios N. Antonoglou, Mario Romandini, Jukka H. Meurman, Markku Surakka, Sok‐Ja Janket, Mariano Sanz

**Affiliations:** ^1^ ETEP Research Group (Etiology and Therapy of Periodontal and Peri‐implant Diseases) Faculty of Odontology, University Complutense of Madrid Madrid Spain; ^2^ Periodontology Unit, Centre for Host Microbiome Interactions Faculty of Dentistry, Oral and Craniofacial Sciences, King's College London London UK; ^3^ Department of Oral and Maxillofacial Diseases University of Helsinki and Helsinki University Hospital Helsinki Finland; ^4^ Department of Oral and Maxillofacial Diseases Kuopio University Hospital Kuopio Finland; ^5^ Center for Clinical and Translational Research The Forsyth Institute Massachusetts Cambridge USA

**Keywords:** all‐cause mortality, CVD mortality, edentulism, longitudinal study, periodontitis, pocket depth

## Abstract

**Aim:**

To investigate the association between periodontitis and edentulism with cardiovascular disease (CVD) and all‐cause mortality.

**Methods:**

Baseline data of 506 subjects including 256 angiographically verified coronary artery disease patients and 250 matched participants in cardiovascular health from the Kuopio Oral Health and Heart study were collected from 1995–1996. Mortality data were accrued until May 31, 2015, and related to baseline periodontal health and edentulism, assessed as exposure and collected by means of clinical and radiographic examination by a single examiner. Cox proportional hazards regression models were fit using covariates such as age, gender, smoking, BMI, and education.

The final sample size for the periodontitis models ranged from 358 to 376, while the edentate models included 413 to 503 subjects for CVD and all‐cause mortality, respectively with no missing values in the predictor, confounders, and outcome.

**Results:**

The strongest association was found between edentulism and CVD and all‐cause mortality (HR: 1.9 ^CVD^, HR: 1.6^all‐cause^; *p* < .01).

**Conclusions:**

Edentulism considered as a poor oral health marker was associated strongly with CVD mortality while periodontitis was not.

## 1 INTRODUCTION

Periodontitis is a chronic infectious‐inflammatory disease associated with dysbiotic dental plaque biofilms characterized by the destruction of the tooth‐supporting tissues (periodontal ligament and alveolar bone).^1^ This disease has a high global prevalence in different populations,^2^, ^3^, ^4^, ^5^, ^6^ and there is mounting evidence that is independently associated with different systemic diseases and co‐morbidities, either directly by bacterial invasion or indirectly through systemic inflammation.^7^, ^8^, ^9^, ^10^, ^11^, ^12^ These include hypertension,^13^ coronary heart disease (CHD)^14^ obesity,^15^ adverse pregnancy outcomes,^16^ diabetes mellitus,^17^, ^18^ and autoimmune diseases such as rheumatoid arthritis^19^ and cancer.^20^, ^21^ Periodontitis has also shown a significant impact in general health care as demonstrated from insurance data^22^ and in patients attending for dental care.^23^ There is also epidemiologic evidence demonstrating that subjects with periodontitis have higher incidence of cardiovascular disease (CVD) events, higher CVD deaths, and higher all‐cause mortality.^9^, ^24^

Although the main cause of tooth loss may vary across countries and populations,^25^ edentulism is the consequence of both dental caries and periodontitis across all age groups globally.^26^, ^27^, ^28^, ^29^, ^30^ Although the rate of tooth loss rises dramatically with advancing age,^31^ in these older populations also caries and especially severe periodontitis have been demonstrated as the main reasons for tooth extraction.^32^ It has also been demonstrated that there is a positive association between periodontitis and all‐cause death and CVD death, as shown in a recent systematic review with meta‐analysis including 57 studies and 5.7 million individuals.^11^ However, this association has not been robustly demonstrated in observational studies, which warrants further confirmation. It was, therefore, the aim of this prospective cohort study to investigate the associations between two oral health conditions (periodontitis and edentulism) and mortality in two cohorts of individuals, namely those with CHD and those without.

## 2 MATERIALS AND METHODS

### 2.1 Ethical and human subjects' protection

This study was approved by the Joint Ethical Committee of the Kuopio University Hospital and the University of Kuopio, and written informed consent was obtained from all participants (KYS19121994/HFO15/94). The longitudinal part of the study was approved by the Boston University Institutional Review Board. This Project adhered to the guidelines set forth by the Declaration of Helsinki and the Belmont Accord to ensure the safety of human research subjects.

### 2.2 Study population

Kuopio Oral Health and Heart (KOHH) study was initiated in 1995–1996 to investigate the association between oral health and angiographically verified CHD patients and non‐CHD individuals in Kuopio, Finland. From this study, we merged the mortality data to the baseline data resulting in 256 CHD patients and 250 age‐ and gender‐matched non‐CHD patient, and a prospective cohort study was conducted.

At baseline, 256 consecutive cardiac patients of any age at the Kuopio University Hospital who were referred for coronary angiography and confirmed as having CHD were invited to participate and be included in the KOHH study. The CHD diagnosis was determined by the presence of at least 50% stenosis in one of the epicardial arteries. Subjects were excluded if they took antibiotics during the previous 30 days or had chronic infection other than dental disease. A total of 250 age‐ and gender‐matched non‐CHD individuals were admitted to the general surgery or otorhinolaryngology (ORL) departments at the same hospital for elective surgery. They were considered as not having CHD based on their medical history and pre‐admission electrocardiogram (ECG). The non‐CHD individuals were recruited from the population within the same geographical area, who would have come to the same hospital had they developed CHD.

The same exclusion and inclusion criteria described above for the CHD patients were applied to the non‐CHD patients. Exclusion criteria were as follows: (1) those who needed emergency coronary by‐pass surgery or valvular replacement surgery; (2) those whose disease status was so grave that a dental examination or dental x‐ray could not be performed safely; and (3) those who required antibiotic prophylaxis prior to periodontal probing. Further details regarding this cohort have been published elsewhere.^14^, ^33^, ^34^, ^35^

### 2.3 Exposure assessment

At the beginning of the study (1995–1996), a single examiner (MS) performed all clinical dental examinations (817) and panoramic tomography examinations (OPGs) using the criteria recommended by the World Health Organization.^7^ The number of teeth included sound or repaired teeth, while non‐restorable root tips were excluded. Dental infections such as periapical lesions that generally signify long‐standing dental caries, pericoronitis defined as infection/inflammation surrounding 3rd molars, (radiolucent follicle around the retained or erupting third molars with diameter >3 mm in the OPGs), or numbers of root remnants with soft tissue inflammation were annotated. Similarly, the amount of vertical bone loss (measured from cementoenamel junction in mm), calculus deposits, and restorations with overhangs were noted. Periodontitis was determined with the community periodontal index of treatment need (CPITN), and if at least two sextants (segments dividing mandible and maxilla into 6) were recorded as having CPITN ≥3 (signifying that sextant had periodontal pocket depth ≥3.5 mm), the patient was coded as having periodontitis. Additional details of dental examinations have been published elsewhere.^34^ Blood tests were performed to evaluate CRP and HDL in batches that included both CHD patients and non‐CHD patients to distribute any potential environmental changes and measurement errors evenly. A high‐sensitivity immunoturbidimetry assay was used to measure CRP with a HITACHI 717 analyzer.

### 2.4 Ascertainment of the endpoints

The outcomes, that is, all‐cause mortality and CVD mortality, were assessed using the mortality records obtained from the Finnish Death Registry in 2008, 2009, 2010 and 2011, and 2015 respectively. The Finnish Office of Statistics collects health data, including mortality, having assigned to each resident of Finland a unique identifier. WHO ICD‐10 codes I00 and I99 were used to identify deaths due to cardiac diseases, chronic ischemic heart disease being the most prevalent (I25). Using a random sample of 100 records, the reliability of the mortality data obtained through ICD‐codes resulted in 99% agreement when comparing with actual physician's diagnosis of death and individual medical records. In a sample of 100 records between 2009 and 2011, we identified three disagreements, but upon further detailed investigation, all turned out to be in agreement. Thus, although ICD‐10 codes were different in these cases, they described the same pathology with different codes. Thus, validity was judged to be 100%.

### 2.5 Statistical analysis

Using R language, a summary (mean, median, min, max) of the populations' characteristics such as mean age, gender, smoking status, body mass index, number of teeth, and dyslipidemia subgroup levels were segmented depending on the presence of edentulism and periodontitis. Additionally, a classification subgrouping CVD‐related causes of death was created in 5 new categories (i.e., 0: no CVD, 1: atherosclerotic heart disease, 2: myocardial infarction, 3: cerebrovascular disease, 4: other CVD).

To estimate the mortality throughout the study, we developed Cox proportional hazards regression models that were fit and adjusted for known covariates. The association between the two oral conditions, edentulism and periodontitis, and the two death outcomes (CVD and all‐cause mortality) was adjusted for using additional sets of covariates such as age, gender, smoking, education, and BMI.

The observations were omitted from an analysis when it contained one or more missing values in the variables being analyzed. Additional measures for the goodness of fit of the regression models were maximum possible *R* ^2^ and R^2^.

In addition, Kaplan–Meier curves were constructed to visually represent survival curves. These Kaplan–Meier curves were plotted based on different stratification according to the diagnosis of CVD and oral health status. First, stratification was performed by the covariates of interest which were edentulism and periodontitis. Then, additional stratification was added, taking into consideration the CVD diagnosis and comparing edentulous vs. dentate patients and patients with or without periodontitis. To estimate the effect of exposure from periodontitis, only dentate individuals were considered and the time variable was expressed as number of days. Secondarily, curves were plotted using the age of the participants as a time scale. Specifically, the age above 65 was used as the time scale with participants contributing in a different fashion in these analyses.^36^

## 3 RESULTS

The demographic characteristics of the patients, smoking, prevalence of CVD, number of teeth (as a factor variable), CVD mortality, all‐cause mortality, and the distribution of deaths by the different CVD groups are shown in Tables 1 and 2. The correlation between periodontitis and the studied variables of interest is provided in Appendix S1. In Table 1, patient characteristics were calculated based on edentulism and in Table 2 based on periodontal health, respectively. In Table 1 (edentulous vs dentate), statistically significant differences were found in some of the variables such as age, gender, education, CHD status, diabetes mellitus, and number of teeth. The general characteristics of the participants defined by their body mass index (BMI), smoking, education, and gender did not differ significantly between subjects with or without periodontitis, although age showed statistically significant differences between the two groups (Tables 2). The main reasons for extractions were caries (59.5%), and periodontitis (31.5%), while 9% of extractions were due to other reasons, as trauma or for esthetic. Additional variables that were explored included CRP levels with median values in no periodontitis patients of 6 mg/L (IQR: 4–10 mg/L), while in periodontitis patients 5 mg/L (IQR: 3–10 mg/L). For dentate, the levels were 5 mg/L (IQR: 3–10 mg/L) and for edentulous 9 mg/L (IQR: 5–15 mg/L). None of these differences showed statistical significance.

**TABLE 1:** Population characteristics based on edentulism status

	Dentate (*N* = 376)	Edentate (*N* = 127)	Overall (*N* = 506)	*p*‐value
Age				<.001
Mean (SD)	58.1 (9.35)	64.7 (8.43)	59.8 (9.54)	
Median [Min, Max]	59.0 [22.0, 79.0]	65.0 [40.0, 87.0]	61.0 [22.0, 87.0]	
Gender				<.001
Male	257 (68.4%)	63 (49.6%)	322 (63.6%)	
Female	119 (31.6%)	64 (50.4%)	184 (36.4%)	
Education				<.001
Mean score (SD)	12.0 (3.11)	10.0 (2.49)	11.5 (3.08)	
Median [Min, Max]	11.0 [6.00, 26.0]	11.0 [6.00, 17.0]	11.0 [6.00, 26.0]	
Smoking				.277
Never	250 (66.5%)	78 (61.4%)	331 (65.4%)	
Current	40 (10.6%)	10 (7.9%)	50 (9.9%)	
Past	77 (20.5%)	33 (26.0%)	110 (21.7%)	
Missing	9 (2.4%)	6 (4.7%)	15 (3.0%)	
CHD				<.001
Undiagnosed	212 (56.4%)	37 (29.1%)	250 (49.4%)	
Diagnosed	164 (43.6%)	90 (70.9%)	256 (50.6%)	
Diabetes mellitus				.0243
Undiagnosed	331 (88.0%)	99 (78.0%)	433 (85.6%)	
Diagnosed	30 (8.0%)	20 (15.7%)	50 (9.9%)	
Missing	15 (4.0%)	8 (6.3%)	23 (4.5%)	
BMI				.385
Mean (SD)	25.1 (3.58)	24.8 (3.95)	25.1 (3.66)	
Median [Min, Max]	24.7 [16.9, 44.4]	24.5 [16.7, 40.9]	24.6 [16.7, 44.4]	
Missing	18 (4.8%)	10 (7.9%)	28 (5.5%)	
N teeth				<.001
Nteeth = 0	0 (0%)	127 (100%)	127 (25.1%)	
0 < Nteeth ≤10	127 (33.8%)	0 (0%)	127 (25.1%)	
10 < Nteeth ≤20	139 (37.0%)	0 (0%)	139 (27.5%)	
Nteeth >20	110 (29.3%)	0 (0%)	110 (21.7%)	
Missing	0 (0%)	0 (0%)	3 (0.6%)	
CVD mortality				<.001
Alive	320 (85.1%)	82 (64.6%)	404 (79.8%)	
Dead	56 (14.9%)	45 (35.4%)	102 (20.2%)	
All‐cause mortality				<.001
Alive	277 (73.7%)	62 (48.8%)	341 (67.4%)	
Dead	99 (26.3%)	65 (51.2%)	165 (32.6%)	
CVD mortality groups				.088
No CVD	52 (13.8%)	29 (22.8%)	81 (16.0%)	
Atherosclerotic heart disease	18 (4.8%)	11 (8.7%)	30 (5.9%)	
Myocardial infarction	15 (4.0%)	4 (3.1%)	19 (3.8%)	
Cerebrovascular disease	5 (1.3%)	9 (7.1%)	14 (2.8%)	
Other CVD	8 (2.1%)	9 (7.1%)	17 (3.4%)	
Alive	278 (73.9%)	65 (51.2%)	345 (68.2%)	

**TABLE 2:** Population characteristics based on periodontal status

	No periodontitis (*N* = 290)	Periodontitis (*N* = 86)	Overall (*N* = 376)	*p*‐value
Age				.0276
Mean (SD)	57.6 (9.46)	60.0 (8.75)	58.1 (9.35)	
Median [Min, Max]	59.0 [22.0, 79.0]	61.0 [42.0, 79.0]	59.0 [22.0, 79.0]	
Gender				.547
Male	201 (69.3%)	56 (65.1%)	257 (68.4%)	
Female	89 (30.7%)	30 (34.9%)	119 (31.6%)	
Education				.24
Mean (SD)	11.9 (3.23)	12.3 (2.64)	12.0 (3.11)	
Median [Min, Max]	11.0 [6.00, 26.0]	12.0 [6.00, 18.0]	11.0 [6.00, 26.0]	
Smoking				.451
Never	190 (65.5%)	60 (69.8%)	250 (66.5%)	
Current	29 (10.0%)	11 (12.8%)	40 (10.6%)	
Past	63 (21.7%)	14 (16.3%)	77 (20.5%)	
Missing	8 (2.8%)	1 (1.2%)	9 (2.4%)	
CHD				.215
Undiagnosed	158 (54.5%)	54 (62.8%)	212 (56.4%)	
Diagnosed	132 (45.5%)	32 (37.2%)	164 (43.6%)	
Diabetes mellitus				.222
Undiagnosed	259 (89.3%)	72 (83.7%)	331 (88.0%)	
Diagnosed	20 (6.9%)	10 (11.6%)	30 (8.0%)	
Missing	11 (3.8%)	4 (4.7%)	15 (4.0%)	
BMI				.34
Mean (SD)	25.0 (3.56)	25.5 (3.64)	25.1 (3.58)	
Median [Min, Max]	24.6 [16.9, 44.4]	24.8 [19.3, 40.0]	24.7 [16.9, 44.4]	
Missing	14 (4.8%)	4 (4.7%)	18 (4.8%)	
Number of teeth				.187
0–10	105 (36.2%)	22 (25.6%)	127 (33.8%)	
10 20	103 (35.5%)	36 (41.9%)	139 (37.0%)	
Nteeth >20	82 (28.3%)	28 (32.6%)	110 (29.3%)	
CVD mortality				1
Alive	247 (85.2%)	73 (84.9%)	320 (85.1%)	
Dead	43 (14.8%)	13 (15.1%)	56 (14.9%)	
All‐cause mortality				.811
Alive	215 (74.1%)	62 (72.1%)	277 (73.7%)	
Dead	75 (25.9%)	24 (27.9%)	99 (26.3%)	
CVD mortality groups				.766
No CVD	39 (13.4%)	13 (15.1%)	52 (13.8%)	
Atherosclerotic heart disease	12 (4.1%)	6 (7.0%)	18 (4.8%)	
Myocardial infarction	13 (4.5%)	2 (2.3%)	15 (4.0%)	
Cerebrovascular disease	4 (1.4%)	1 (1.2%)	5 (1.3%)	
Other CVD	6 (2.1%)	2 (2.3%)	8 (2.1%)	
Alive	216 (74.5%)	62 (72.1%)	278 (73.9%)	

From the 127 edentulous patients, only 51.2% survived through the total follow‐up period. Among the 65 deaths in the edentulous group, 45 (69%) were due to a CVD. From the 86 patients with periodontitis, 72.1% survived throughout the same period. In this group, there were a total of 24 deaths, 13 (54%) due to CVD (Table 2).

### 3.1 Survival and regression analyses without taking into account the CHD diagnoses

**FIGURE 1:**
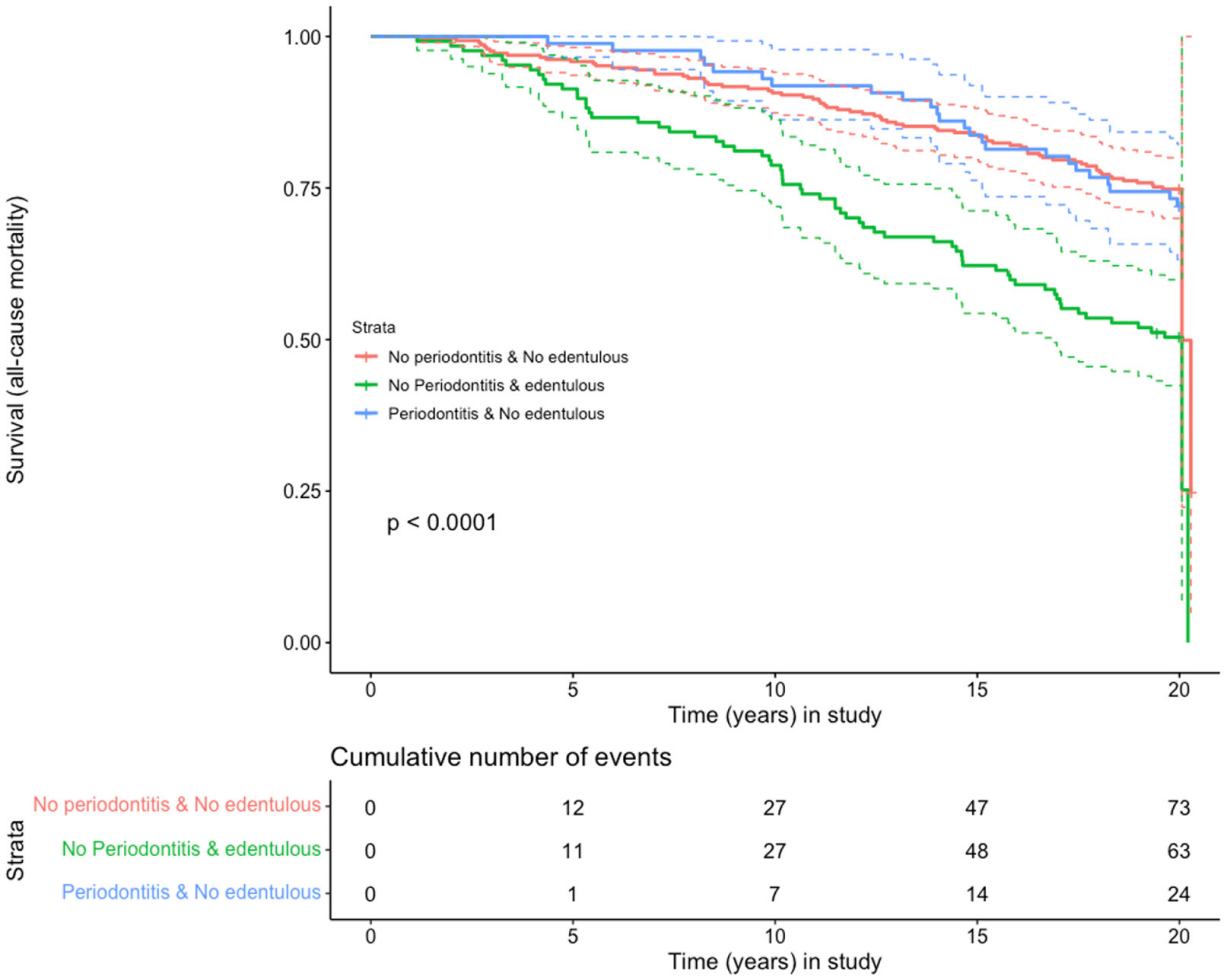
Exposures: (A) dentate with no periodontitis; (B) edentulous; (C) periodontitis Outcome: all‐cause mortality; Time: years in the study

**FIGURE 2:**
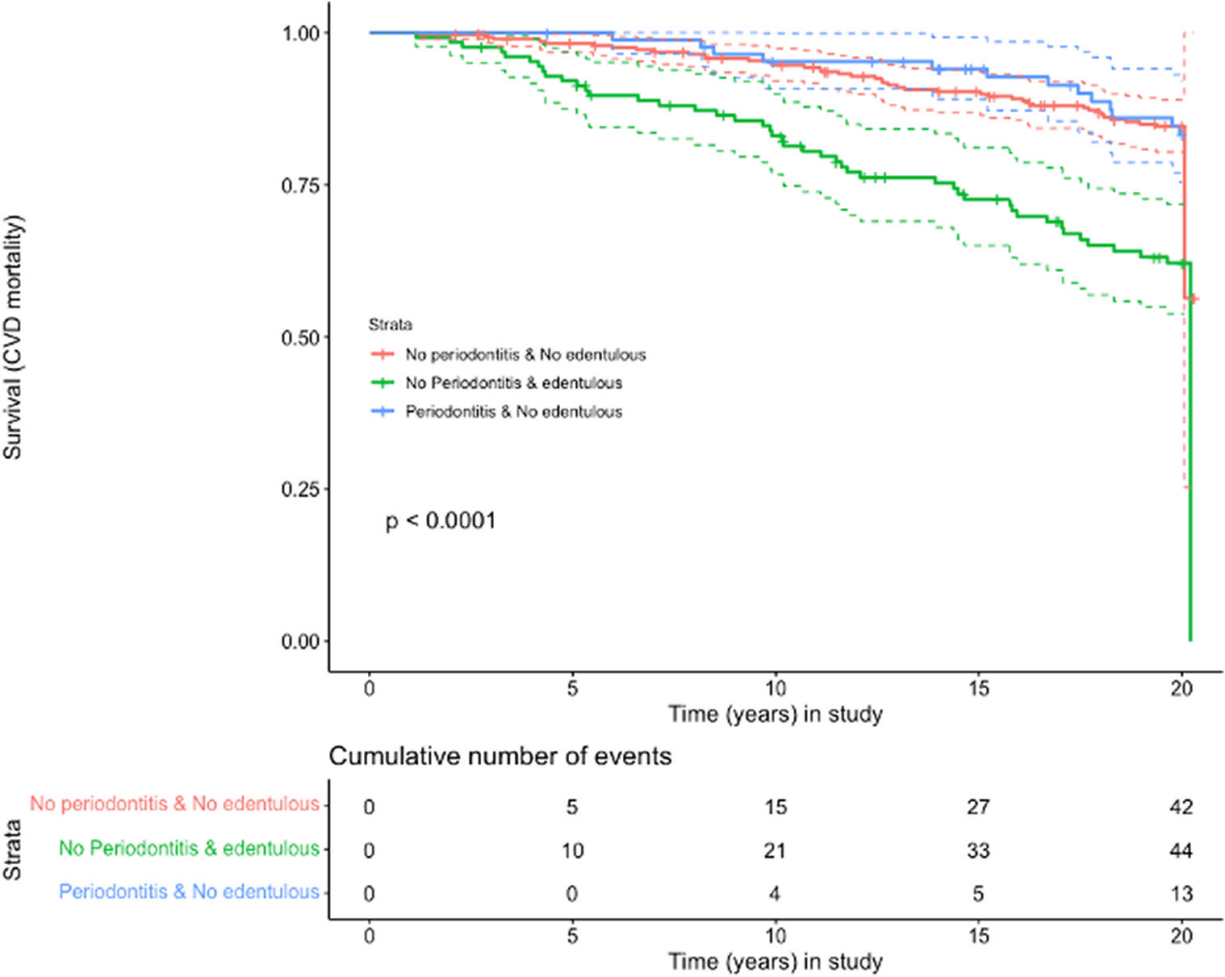
Exposures: (A) dentate with no periodontitis; (B) edentulous; (C) periodontitis Outcome: CVD mortality; Time: years in the study

The Cox proportional hazard models with edentulism as the main exposure (Tables 3 and 4) showed increased all‐cause and CVD mortalities. In these survival curves, a constant statistically significant effect of edentulism was seen for both all‐cause and CVD mortality (*p* < .001) (Figures 1 and 2). Additional Cox models including potential explanatory variables such as gender, age, BMI, smoking, and education showed significant associations between edentulism and CVD mortality, being the hazard ratio of 1.9 (95% CI 1.4–2.4). The corresponding Cox model for edentulism and all‐cause mortality also showed statistically significant effect with an HR of 1.4 (1.6, 95% CI = 1.2–1.9).

**TABLE 3:** Cox proportional hazard models; Dependent variable: cardiovascular mortality.Independent variable: edentulism

	(1)	(2)	(3)	(4)	(5)	(6)
Edentulism	3.059***	1.747***	2.048***	1.783**	1.752**	1.868***
	(0.200)	(0.211)	(0.216)	(0.227)	(0.237)	(0.242)
Age		1.124***	1.126***	1.126***	1.125***	1.120***
		(0.016)	(0.015)	(0.016)	(0.016)	(0.016)
Sex			0.418***	0.656	0.654	0.681
			(0.222)	(0.271)	(0.271)	(0.278)
Smoking				1.586***	1.579***	1.574***
				(0.128)	(0.129)	(0.133)
Education					0.990	0.978
					(0.038)	(0.039)
Bmi						0.981
						(0.035)
Observations	440	440	440	427	427	413
*R* ^2^	0.063	0.197	0.227	0.241	0.242	0.241
Max. Possible *R* ^2^	0.935	0.935	0.935	0.925	0.925	0.919

*Note:* *p < .1; **p < .05; ***p < .01.

**TABLE 4:** Cox proportional hazard models; Dependent variable: all‐cause mortality Independent variable: edentulism

	(1)	(2)	(3)	(4)	(5)	(6)
Edentulism	3.059***	1.464**	1.654***	1.485**	1.495**	1.551**
	(0.200)	(0.166)	(0.170)	(0.178)	(0.185)	(0.187)
Age		1.111***	1.112***	1.113***	1.113***	1.112***
		(0.012)	(0.011)	(0.012)	(0.012)	(0.012)
Sex			0.471***	0.622**	0.623**	0.651**
			(0.176)	(0.205)	(0.205)	(0.208)
Smoking				1.402***	1.405***	1.402***
				(0.098)	(0.098)	(0.099)
Education					1.004	0.998
					(0.028)	(0.029)
Bmi						1.006
						(0.025)
Observations	440	503	503	488	488	474
*R* ^2^	0.063	0.219	0.249	0.260	0.260	0.260
Max. Possible *R* ^2^	0.935	0.980	0.980	0.978	0.978	0.977

*Note:* *p < .1; **p < .05; ***p < .01.

### 3.2 Survival and regression analyses taking into account CHD diagnoses

The burdening effect of CHD diagnosis and edentulism combined in the survival curves was evident for CVD mortality and for all‐cause mortality Appendix S5 and Appendix S7 (*p* < .01). In these regression models (Appendix S11), the effect for CVD mortality was statistically significant after adjusting for age, gender, smoking, BMI, and education with HR 1.9 (95% CI 1.2–2.5) and also for all‐cause mortality (Appendix S12) with HR 1.8 (95% CI 1.2–2.3).

The same effect considering periodontitis as main exposure did not show statistical significance (Appendix S13 and S14).

### 3.3 Survival analyses considering age above 65 years as the time scale

**FIGURE 3:**
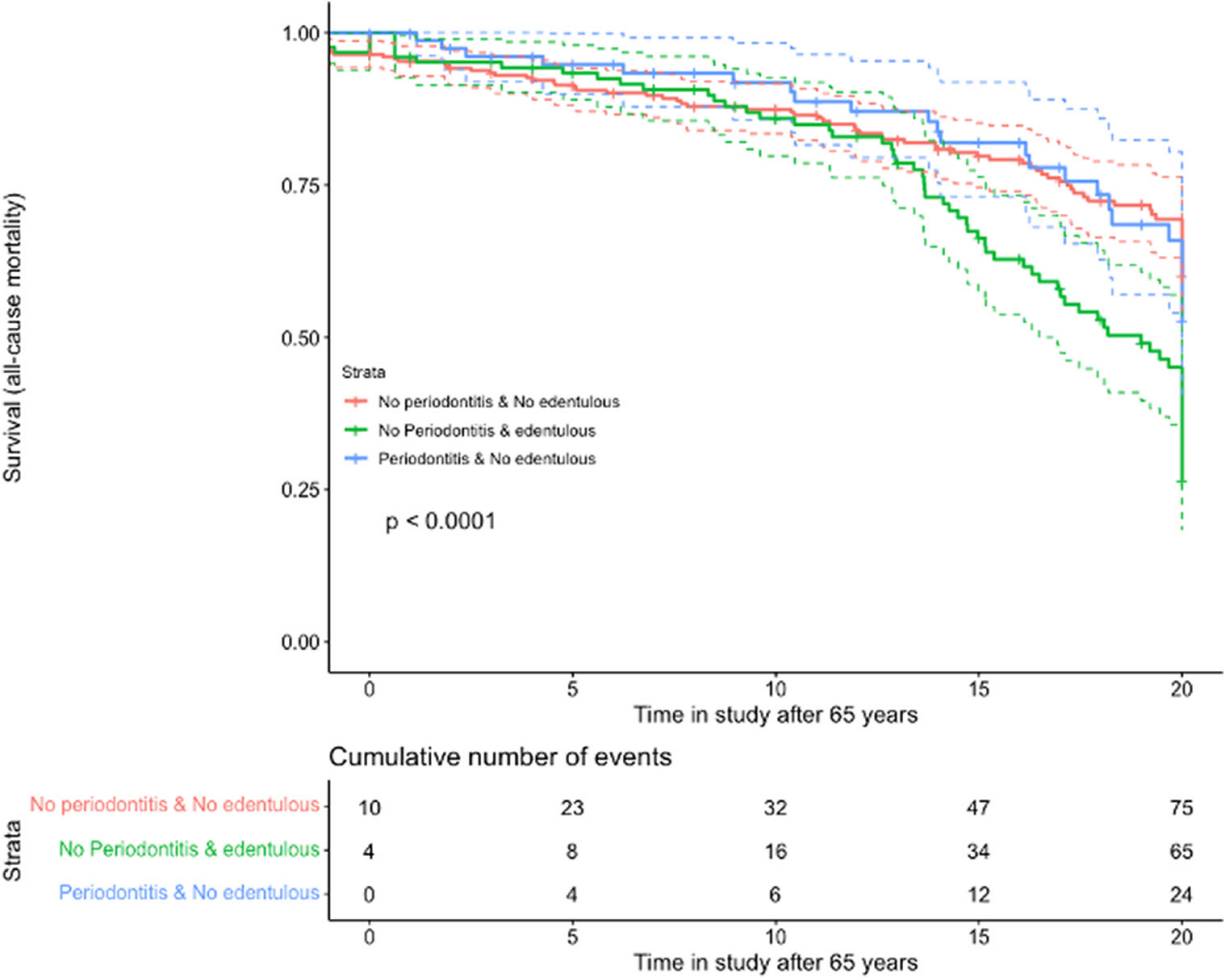
Exposures: (A) dentate with no periodontitis; (B) edentulous; (C) periodontitis Outcome: all‐cause mortality; Time: years after 65 in the study

**FIGURE 4:**
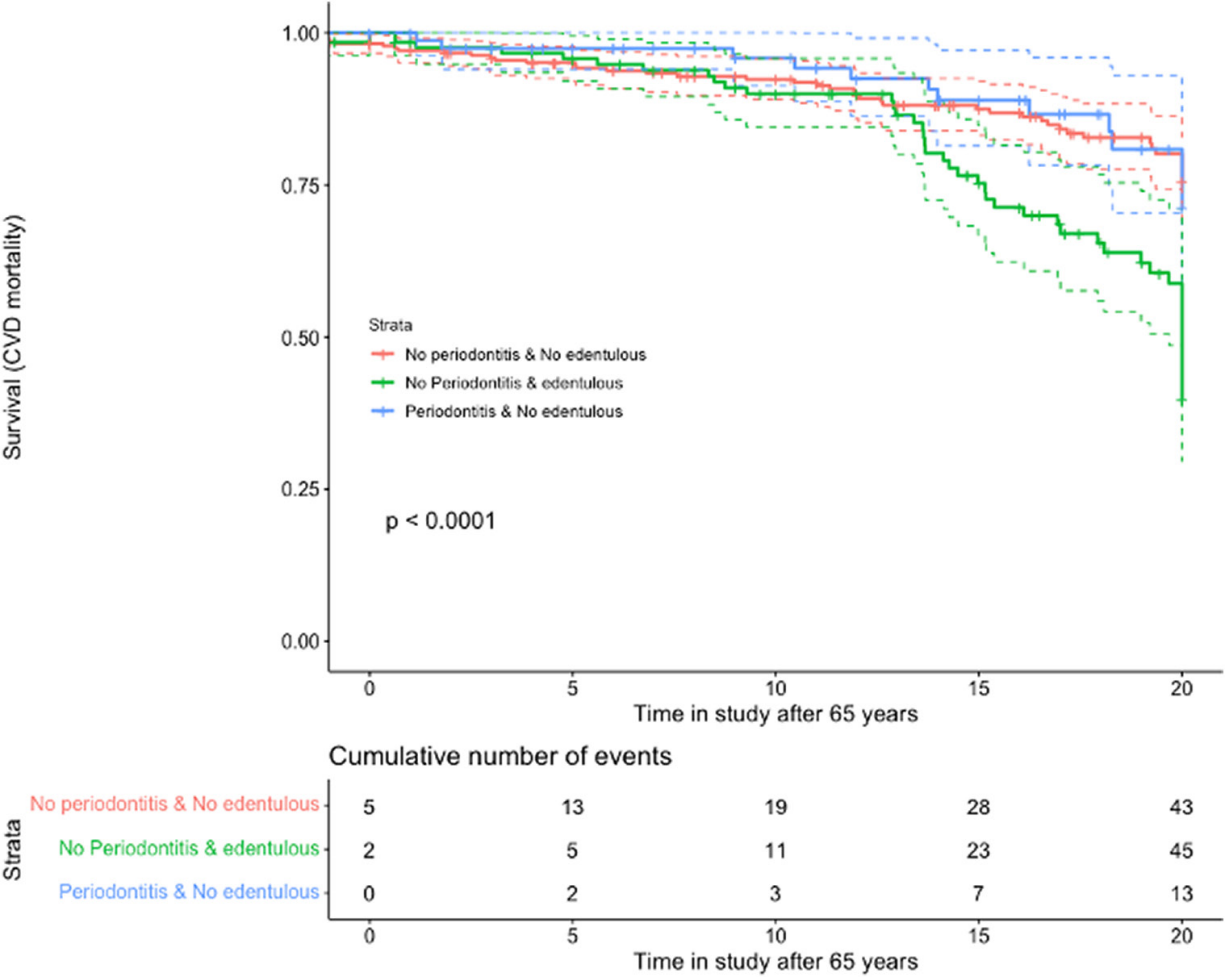
Exposures: (A) dentate with no periodontitis; (B) edentulous; (C) periodontitis Outcome: CVD mortality; Time: years after 65 in the study

Similarly to explore the effect of increasing age we drew additional survival curves (Figures 3 and 4), and to explore the exposure of severe periodontitis and CVD diagnosis for all‐cause or CVD mortality, although this exposure, however, did not show any significant effect (Appendix S6, S7). A final model to explore the effect of severe periodontitis on CVD mortality also failed to show significant effects after adjusting for potential confounding factors. In fact, these effects were largest when the crude model was only adjusted for CHD (Appendix S10; HR: 1.2, 95% CI 0.5–2.6). Among the factors affecting the relationship between periodontitis and CVD mortality as positive confounders enhancing the effects of periodontitis, we identified the baseline CHD, past smoking and gender, and as negative confounders mitigating the effect of periodontitis, we identified age, fibrinogen levels, and diabetes. Among these, only baseline CHD, age, fibrinogen levels, and gender emerged as significant determinants of CHD mortality.

## 4 DISCUSSION

In the present study, the association between periodontitis and edentulousness and mortality (all‐cause and CVD mortality) was investigated using a cohort study design. The effect of edentulousness was stronger than the effect of periodontitis, being statistically significant after adjusting for age, gender, BMI, education, and smoking. This effect seemed that it was also significant when studied with a Cox proportional hazard model and after stratifying for previous CVD diagnosis (Appendix S2 and S3). Severe periodontitis, however, did not seem to exert a similar effect on patient mortality.

Similar to the results from this investigation, a large study of 57 001 women in the context of “Women's Health Initiative Observational Study” showed a clear association between edentulism and increased risk of CVD and total mortality.^24^ Different studies have also reported that tooth loss and edentulousness are significant factors for all‐cause mortality, especially in older individuals^11^, ^37^ and also when comparing non‐rehabilitated vs rehabilitated edentulism in their association with mortality.^38^ Additional factors and comorbidities have also been linked to the edentulous state,^39^ including smoking, presence of CVD, osteoporosis, neuropathy, dementia, and rheumatoid arthritis.^40^ Other causes of mortality, such as cancer, have also been recently associated with edentulousness using the database from the National Health and Nutrition Examination Survey III.^41^ In fact, a significant association between pancreatic cancer (PC) and both periodontitis and edentulism has been reported,^42^ The explanation for these associations, and their likely temporal sequence, may be heterogeneous and in part due to the impact of periodontitis, which may cause tooth loss, hence ensuing partial or even complete edentulousness. For instance, in diabetic patients there is a significant association between periodontitis and extensive tooth loss which may be the consequence of inadequate glycemic control. The term diabetes tooth loss has been recently introduced and refers to the loss of teeth that is directly linked to diabetes.^43^, ^44^, ^45^, ^46^, ^47^

The effect of periodontitis on all‐cause mortality and CVD mortality was, however, weak and statistically insignificant in this observational study (Figures 1, 2, 3, 4). This could be explained by residual confounding related to other factors, such as socioeconomical, or to a potential misclassification of periodontitis. The combined effects of periodontitis and CHD seemed to show a trend of an association with mortality, but due to the clear effect of CHD alone, as can be clearly seen on the corresponding survival curves, the role of periodontitis could not be appraised (Appendix S2,S3 and S6,S7).

However, in spite of the lack of a significant effect of periodontitis in mortality reported in this study, periodontitis has been significantly associated with a large number of systemic diseases, mainly those having chronic inflammation as the main pathophysiology, such as atherosclerosis‐related CVDs, diabetes, Alzheimer's diseases, pneumonia, rheumatoid arthritis, intestinal inflammation, chronic liver disease, and cancer.^48^ This evidence from observational studies has not been substantiated for most of these intervention studies.^49^

One of the strengths of the present study are its prospective design and long follow‐up (20+ years). Additional strengths include the collection of both dental and medical data with well‐validated methods. The long‐term follow‐up particularly allowed for studying the effects of additional conditions influencing the outcomes, such as the CHD diagnosis.

The present study also has clear limitations, since periodontitis was determined indirectly by the bone loss shown in the radiographs, which may limit the specificity of determining appropriately the exposure. In addition, baseline data were collected once, there is no information on the rate of periodontitis progression for each individual, which may in part explain the lack of a significant association between periodontal status and mortality. Also, there is limited information of other important confounding factors that could have influenced this association. Nonetheless, our population risk estimates in simulated data appear to support the generalizability of our results.^22^

## 5 CONCLUSION

Based on the present findings, and after adjusting for confounding factors, this study has shown that edentulousness but not periodontitis increased both the CVD and all‐cause mortality. Unequivocal clinical evidence on the effects of periodontitis on mortality would require precise direct determination of disease severity and progression.

## AUTHOR CONTRIBUTIONS

GNA, MR, SJ, JM, and MS contributed to study conception and study design. MS collected all the data in this study for two decades. GNA, MR, SJ, SM, and MS contributed to data management. GNA, MR, JM, SJ, SM, and MS contributed to data analysis, data interpretation and manuscript drafting. GNA, MR, JM, SJ, SM, and MS contributed to critical revision of the manuscript. All authors approved the final version.

## FUNDING INFORMATION

None.

## CONFLICT OF INTEREST

None.

## Supporting information


Appendix S1–S14

Appendix S1–S8

Data S1

Data S2

## Data Availability

The data that support the findings of this study are openly available in Mendeley Data at https://data.mendeley.com, reference number 10.17632/z8ffdjdxyk.1.
