# Supplementary material for: Periodontitis and edentulism as risk indicators for mortality: Results from a prospective cohort study with 20 years of follow‐up
Source: J Periodontal Res. 2022 Oct 25;58(1):12–21. doi: 10.1111/jre.13061 (PMC10092146; doi:10.1111/jre.13061)
Supplement: Supplementary file 1 — Appendix S1–S14 [file JRE-58-12-s002.docx]

Appendix 1


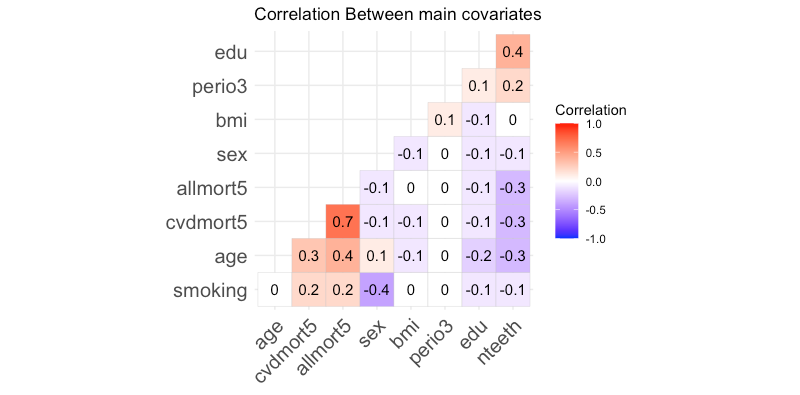


Appenidx 2.


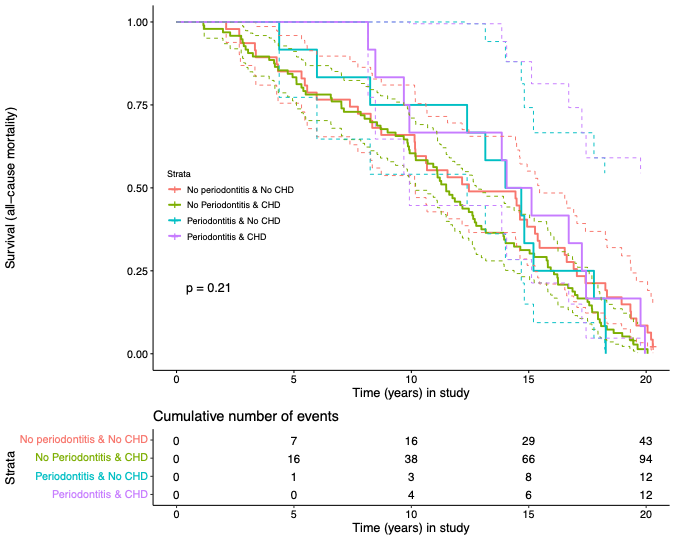


Appendix 3.


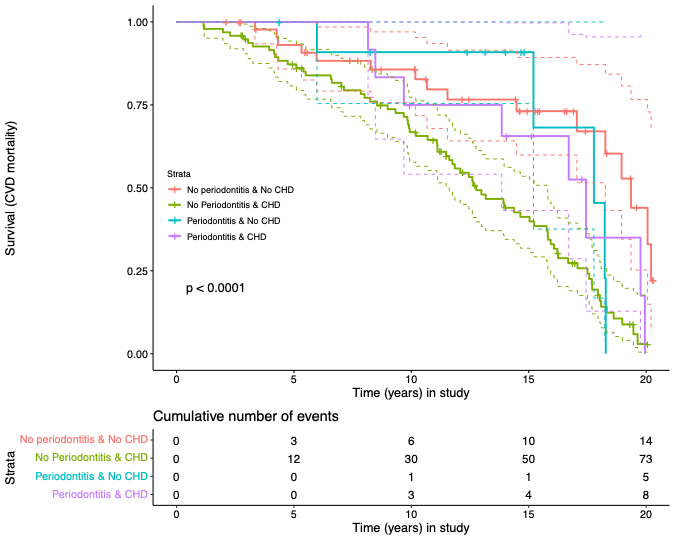


Appendix 4.


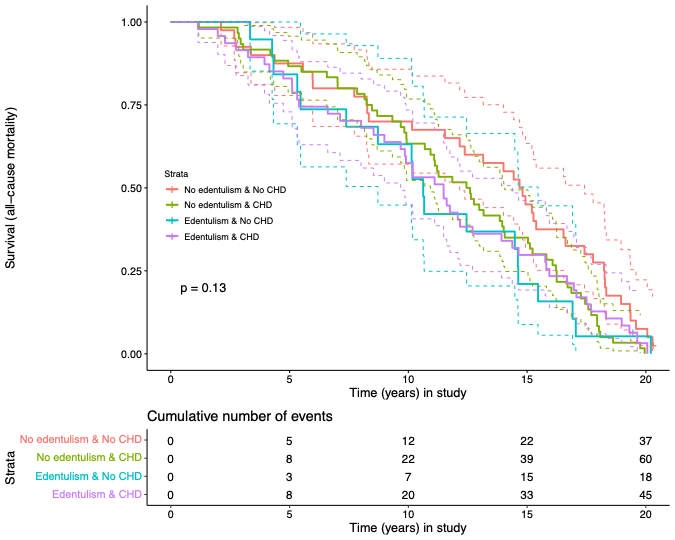


Appendix 5.


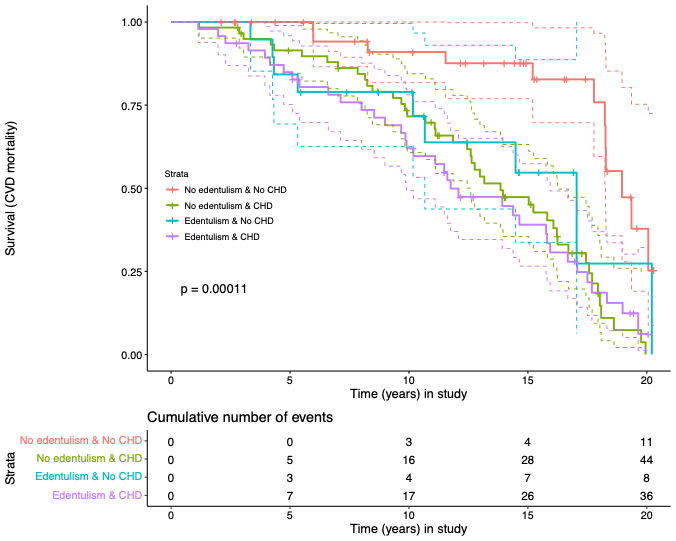


Appendix 6.


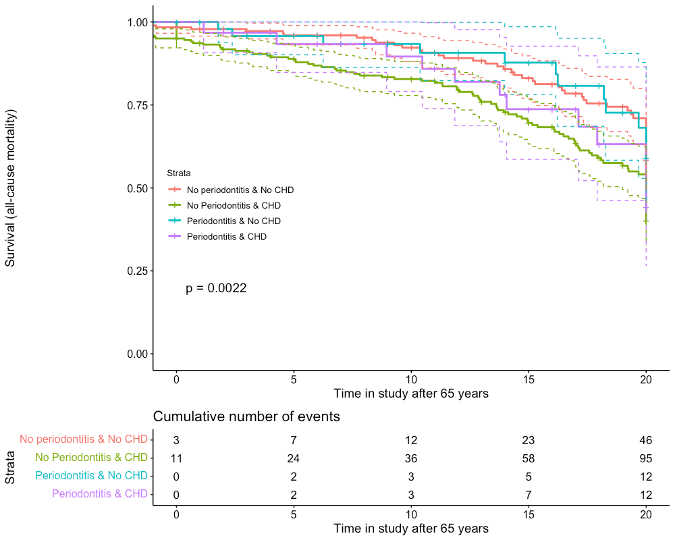


Appendix 7.


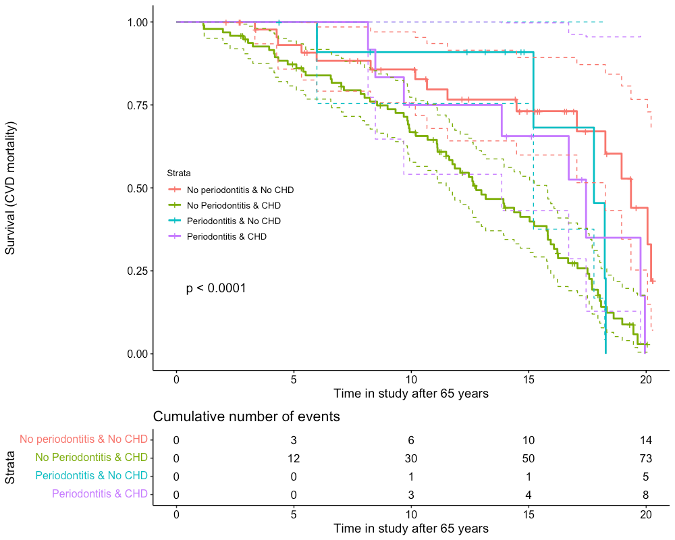


Appendix 8.


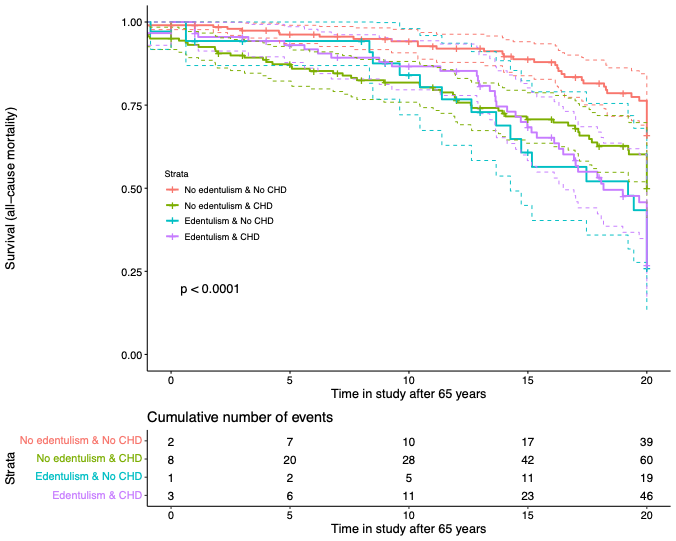


Appendix 9.


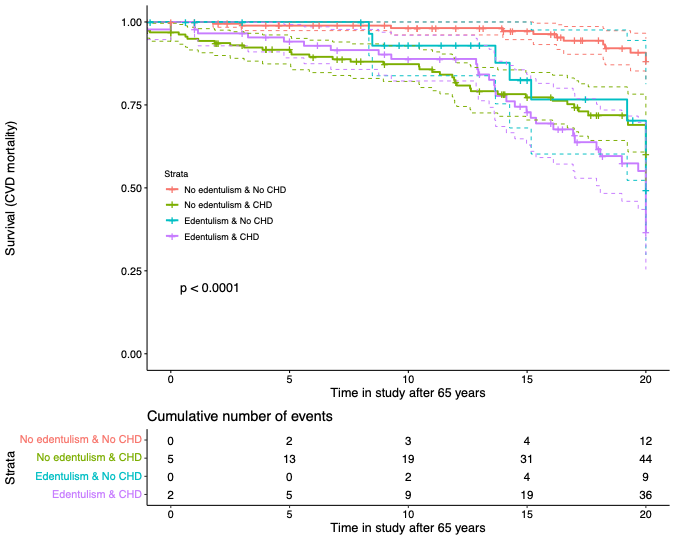


Appendix 11.

Appendix 12.

Appendix 13.

Appendix 14.
