# Supplementary material for: Periodontitis and edentulism as risk indicators for mortality: Results from a prospective cohort study with 20 years of follow‐up
Source: J Periodontal Res. 2022 Oct 25;58(1):12–21. doi: 10.1111/jre.13061 (PMC10092146; doi:10.1111/jre.13061)
Supplement: Supplementary file 2 — Appendix S1–S8 [file JRE-58-12-s001.docx]

Appendix 1.


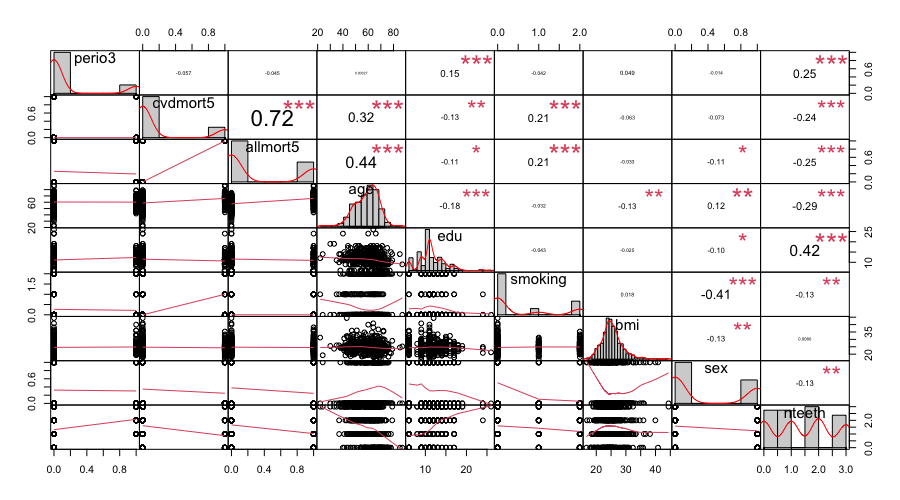


Appendix 2.

Exposures: a) CHD+ diagnosis and edentulous; b) CHD- diagnosis OR no edentulous

Outcome: all-cause mortality; Time: number of days


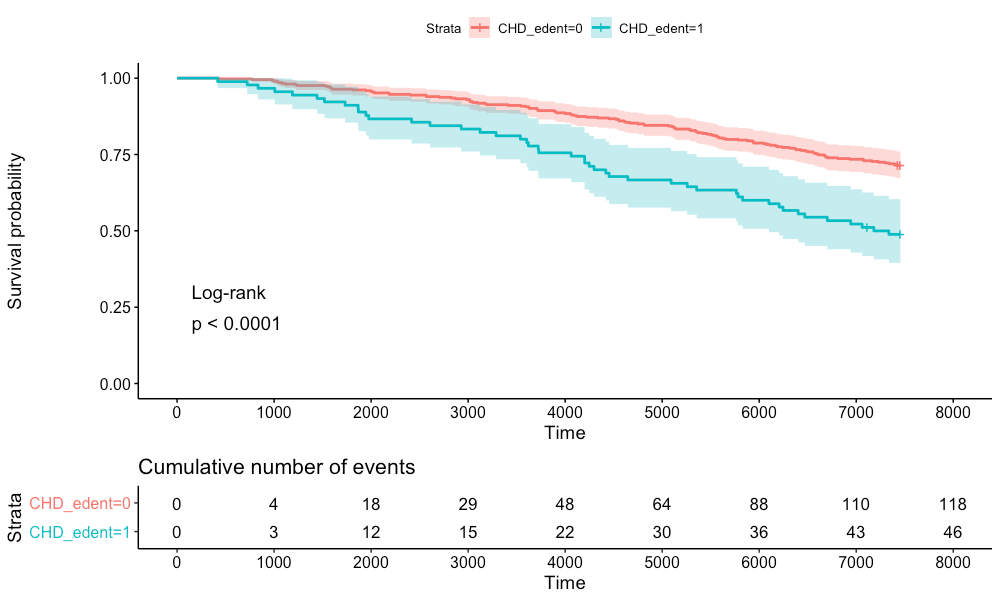


CHD diag- OR no edent

CHD diag+ & edent

Appendix 3.

Exposures: a) CHD+ diagnosis and edentulous; b) CHD- diagnosis OR no edentulous

Outcome: CVD mortality; Time: number of days


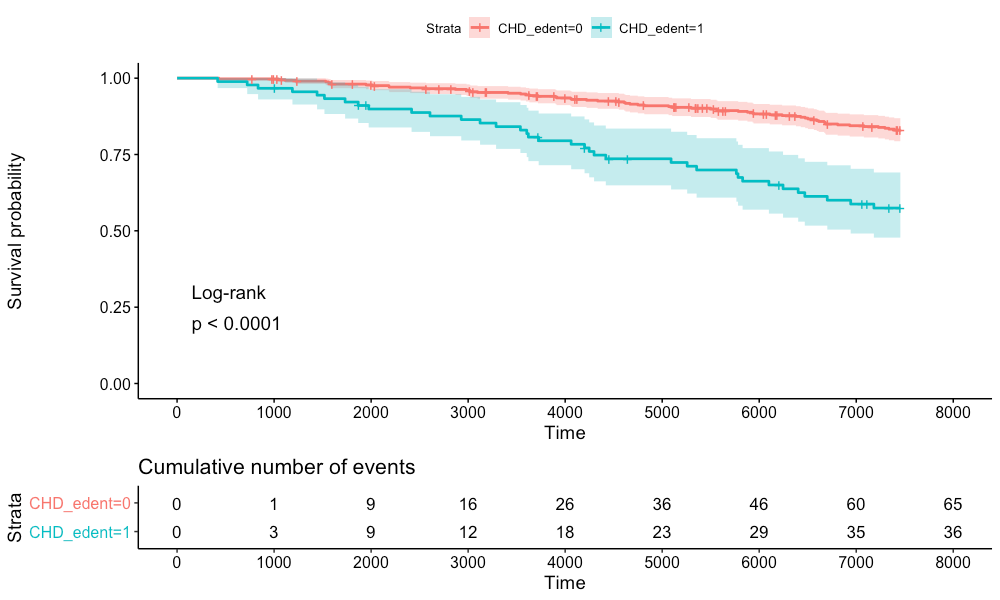


CHD diag- OR no edent

CHD diag+ & edent

Appendix 4.

Exposures: a) CHD+ diagnosis and periodontitis; b) CHD- diagnosis OR no periodontitis

Outcome: all-cause mortality; Time: number of days


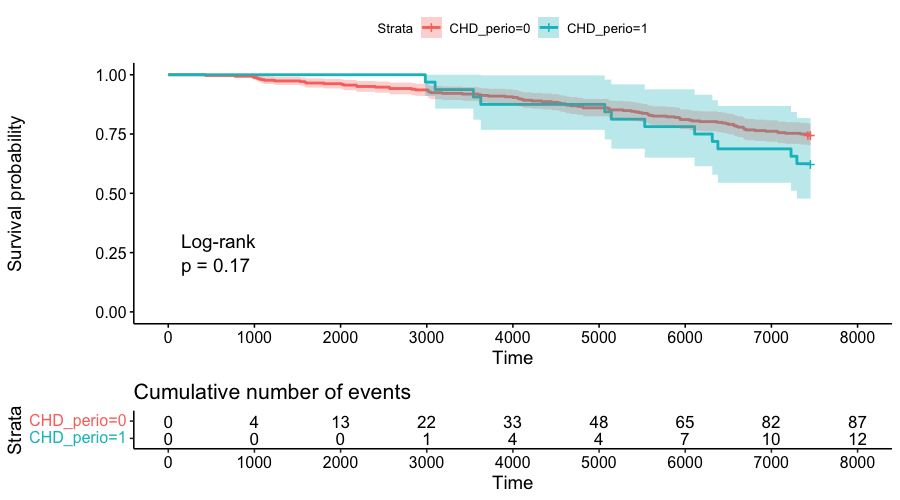


CHD diag- OR no perio

CHD diag+ & perio

Appendix 5.

Exposures: a) CHD+ diagnosis and periodontitis b) CHD- diagnosis OR no periodontitis


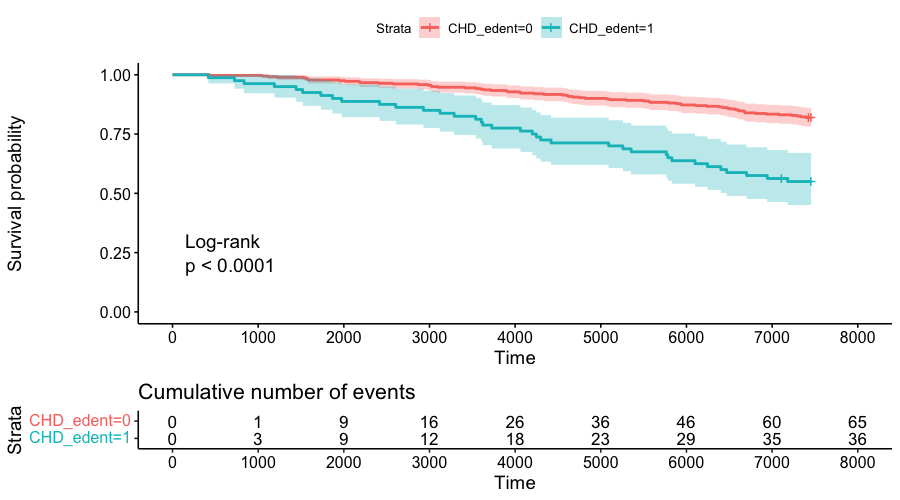
Outcome: CVD mortality; Time: number of days

CHD diag- OR no perio

CHD diag+ & perio

| Appendix 6. Cox proportional hazard models;  Dependent variable: cardiovascular mortality.  Independent variable: periodontitis | | | | | | |
| --- | --- | --- | --- | --- | --- | --- |
|  | (1) | (2) | (3) | (4) | (5) | (6) |
|  | | | | | | |
| perio3 | 1.017 | 0.833 | 0.893 | 0.980 | 0.998 | 1.004 |
|  | (0.317) | (0.318) | (0.319) | (0.324) | (0.327) | (0.340) |
|  |  |  |  |  |  |  |
| age |  | 1.107^***^ | 1.118^***^ | 1.127^***^ | 1.126^***^ | 1.118^***^ |
|  |  | (0.020) | (0.020) | (0.021) | (0.021) | (0.021) |
|  |  |  |  |  |  |  |
| sex |  |  | 0.360^***^ | 0.520^*^ | 0.506^*^ | 0.500^*^ |
|  |  |  | (0.340) | (0.381) | (0.384) | (0.403) |
|  |  |  |  |  |  |  |
| smoking |  |  |  | 1.797^***^ | 1.769^***^ | 1.807^***^ |
|  |  |  |  | (0.156) | (0.159) | (0.165) |
|  |  |  |  |  |  |  |
| edu |  |  |  |  | 0.974 | 0.968 |
|  |  |  |  |  | (0.048) | (0.050) |
|  |  |  |  |  |  |  |
| bmi |  |  |  |  |  | 0.983 |
|  |  |  |  |  |  | (0.051) |
|  |  |  |  |  |  |  |
|  | | | | | | |
| Observations | 333 | 333 | 333 | 325 | 325 | 316 |
| R^2^ | 0.00001 | 0.095 | 0.123 | 0.165 | 0.165 | 0.155 |
| Max. possible R^2^ | 0.854 | 0.854 | 0.854 | 0.839 | 0.839 | 0.822 |
|  | | | | | | |
|  | ^*^p<0.1; ^**^p<0.05; ^***^p<0.01 | | | | | |

| Appendix 7. Cox proportional hazard models;  Dependent variable: all-cause mortality.  Independent variable: periodontitis | | | | | | |
| --- | --- | --- | --- | --- | --- | --- |
|  | (1) | (2) | (3) | (4) | (5) | (6) |
|  | | | | | | |
| perio3 | 1.064 | 0.854 | 0.869 | 0.941 | 0.943 | 0.962 |
|  | (0.235) | (0.236) | (0.237) | (0.239) | (0.240) | (0.245) |
|  |  |  |  |  |  |  |
| age |  | 1.111^***^ | 1.116^***^ | 1.122^***^ | 1.122^***^ | 1.120^***^ |
|  |  | (0.015) | (0.014) | (0.015) | (0.015) | (0.015) |
|  |  |  |  |  |  |  |
| sex |  |  | 0.478^***^ | 0.591^**^ | 0.589^**^ | 0.597^*^ |
|  |  |  | (0.239) | (0.264) | (0.267) | (0.273) |
|  |  |  |  |  |  |  |
| smoking |  |  |  | 1.510^***^ | 1.508^***^ | 1.520^***^ |
|  |  |  |  | (0.119) | (0.120) | (0.122) |
|  |  |  |  |  |  |  |
| edu |  |  |  |  | 0.997 | 0.996 |
|  |  |  |  |  | (0.034) | (0.035) |
|  |  |  |  |  |  |  |
| bmi |  |  |  |  |  | 1.016 |
|  |  |  |  |  |  | (0.034) |
|  |  |  |  |  |  |  |
|  | | | | | | |
| Observations | 376 | 376 | 376 | 367 | 367 | 358 |
| R^2^ | 0.0002 | 0.158 | 0.181 | 0.210 | 0.210 | 0.204 |
| Max. possible R^2^ | 0.952 | 0.952 | 0.952 | 0.948 | 0.948 | 0.944 |
|  | | | | | | |
|  | ^*^p<0.1; ^**^p<0.05; ^***^p<0.01 | | | | | |

| Appendix 8. Models predicting CVD mortality by periodontitis with significant confounding factors added stepwise | | |
| --- | --- | --- |
| Exposure: periodontitis | Hazard ratio (95% confidence interval) | p-value |
| Model I periodontitis alone | 1.034 (0.485-2.206) | 0.9306 |
| Model II periodontis + baseline CAD | 1.192 (0.540-2.631) | 0.6644 |
| Model III periodontis + CAD+age | 1.011 (0.445-2.298) | 0.9793 |
| Model IV periodontis + CAD+age+ fibrinogen | 0.959 (0.420-2.193) | 0.9215 |
| Model V periodontis + CAD+age+ fibrinogen+ gender | 1.008 (0.432-2.352) | 0.9850 |
| Model VI periodontis + CAD+age+ fibrinogen+ gender + past smoking | 1.036 (0.442-2.430) | 0.9356 |
| Model VII periodontis + CAD + age+ fibrinogen+ gender + past smoking + diabetes | 0.971 (0.407-2.316) | 0.9477 |
